# Insulin resistance, diabetic kidney disease, and all-cause mortality in individuals with type 2 diabetes: a prospective cohort study

**DOI:** 10.1186/s12916-021-01936-3

**Published:** 2021-03-15

**Authors:** Giuseppe Penno, Anna Solini, Emanuela Orsi, Enzo Bonora, Cecilia Fondelli, Roberto Trevisan, Monica Vedovato, Franco Cavalot, Gianpaolo Zerbini, Olga Lamacchia, Antonio Nicolucci, Giuseppe Pugliese, for the Renal Insufficiency And Cardiovascular Events (RIACE) Study Group, Giuseppe Pugliese, Giuseppe Penno, Anna Solini, Enzo Bonora, Emanuela Orsi, Roberto Trevisan, Luigi Laviola, Antonio Nicolucci

**Affiliations:** 1 Department of Clinical and Experimental Medicine, University of Pisa, Pisa, Italy; 2 Department of Surgical, Medical, Molecular and Critical Area Pathology, University of Pisa, Pisa, Italy; 3 Diabetes Unit, IRCCS “Cà Granda - Ospedale Maggiore Policlinico” Foundation, Milan, Italy; 4 Division of Endocrinology, Diabetes and Metabolism, University and Hospital Trust of Verona, Verona, Italy; 5 Diabetes Unit, University of Siena, Siena, Italy; 6 Endocrinology and Diabetes Unit, Azienda Ospedaliera Papa Giovanni XXIII, Bergamo, Italy; 7 Department of Clinical and Experimental Medicine, University of Padua, Padua, Italy; 8 Department of Clinical and Biological Sciences, University of Turin, Orbassano, Italy; 9 Complications of Diabetes Unit, Division of Metabolic and Cardiovascular Sciences, San Raffaele Scientific Institute, Milan, Italy; 10 Department of Medical Sciences, University of Foggia, Foggia, Italy; 11 Centre for Outcomes Research and Clinical Epidemiology (CORESEARCH), Pescara, Italy; 12 Department of Clinical and Molecular Medicine, “La Sapienza” University, Via di Grottarossa, 1035-1039, 00189 Rome, Italy

**Keywords:** Type 2 diabetes, Estimated glucose disposal rate, All-cause mortality, Diabetic kidney disease, Albuminuria, Glomerular filtration rate, Mellitus

## Abstract

**Background:**

It is unclear whether insulin resistance (IR) contributes to excess mortality in patients with type 2 diabetes independent of diabetic kidney disease (DKD), which is strongly associated with IR and is a major risk factor for cardiovascular disease (CVD), the main cause of death in these individuals. We tested this hypothesis in patients with type 2 diabetes from the Renal Insufficiency And Cardiovascular Events Italian Multicentre Study.

**Methods:**

This observational, prospective, cohort study enrolled 15,773 patients with type 2 diabetes attending 19 Italian Diabetes Clinics in 2006–2008. Insulin sensitivity was assessed as estimated glucose disposal rate (eGDR), which was validated against the euglycaemic-hyperinsulinemic clamp technique. Vital status on October 31, 2015, was retrieved for 15,656 patients (99.3%). Participants were stratified by eGDR tertiles from T1 (≥ 5.35 mg/kg/min) to T3 (≤ 4.14 mg/kg/min, highest IR).

**Results:**

CVD risk profile was worse in T2 and T3 vs T1. eGDR tertiles were independently associated with micro- and macroalbuminuria and the albuminuric DKD phenotypes (albuminuria with preserved or reduced estimated glomerular filtration rate [eGFR]) as well as with eGFR categories or the nonalbuminuric DKD phenotype. Over a 7.4-year follow-up, unadjusted death rates and mortality risks increased progressively across eGDR tertiles, but remained significantly elevated after adjustment only in T3 vs T1 (age- and gender- adjusted death rate, 22.35 vs 16.74 per 1000 person-years, *p* < 0.0001, and hazard ratio [HR] adjusted for multiple confounders including DKD, 1.140 [95% confidence interval [CI], 1.049–1.238], *p* = 0.002). However, eGDR was independently associated with mortality in participants with no DKD (adjusted HR, 1.214 [95% CI, 1.072–1.375], *p* = 0.002) and in those with nonalbuminuric DKD (1.276 [1.034–1.575], *p* = 0.023), but not in those with the albuminuric DKD phenotypes. Moreover, the association was stronger in males and in younger individuals and was observed in those without but not with prior CVD, though interaction was significant only for age.

**Conclusions:**

The proxy of insulin sensitivity eGDR predicts all-cause mortality in type 2 diabetes, independent of confounders including DKD. However, the impact of IR in individuals with albuminuric DKD may be mediated by its relationship with albuminuria.

**Trial registration:**

ClinicalTrials.gov, NCT00715481, retrospectively registered 15 July 2008.

**Supplementary Information:**

The online version contains supplementary material available at 10.1186/s12916-021-01936-3.

## Background

Risk of death from any cause and cardiovascular disease (CVD) is significantly higher in patients with type 2 diabetes (T2D) than in people without diabetes [1], though it varies widely from lower risk, approaching that of the general population, to substantial excess of risk, especially in T2D individuals with younger age [2, 3], worse glycaemic control [2], and impaired renal function [2, 3]. In particular, diabetic kidney disease (DKD) is a major contributor to excess mortality in T2D, conferring a very high risk in younger patients and fully accounting for the excess of risk in the older ones [2, 3].

However, in the last decades, a decline in all-cause mortality and in the incidence of CVD has been consistently reported in T2D individuals [4, 5]. Patients with T2D from the Swedish National Diabetes Register showed a ~ 20% greater reduction in overall CVD than controls, although fatal outcomes declined to a lesser extent [4]. Likewise, the analysis of data from the National Health Interview Survey Linked Mortality files showed that, among US adults with diabetes, death from any cause declined by 20%, whereas death from CVD decreased by 32% every 10 years. Death rates declined also among nondiabetic individuals, but reductions were significantly greater among people with diabetes, so that differences in all-cause and CVD mortality between individuals with and without diabetes were reduced by about a half [5].

Intensified, multifactorial, target-driven treatment has substantially contributed to the decline in mortality in people with T2D, by increasing the years of life gained and the time free from incident CVD [6] as well as by slowing progression of DKD toward end-stage renal disease [7]. Nevertheless, T2D persons still have a large excess in total and CVD mortality suggesting that other risk factors are involved [2].

Insulin resistance (IR) is associated with an increased risk of CVD in people with T2D [8, 9] and also in those with type 1 diabetes (T1D) [10]. Other than clustering with hyperglycaemia, dyslipidaemia, hypertension, and obesity, which are targeted by multifactorial interventions, IR is associated with endothelial dysfunction [11] and a pro-oxidant, pro-inflammatory, and pro-coagulant environment [12]. In addition, IR is strongly related to DKD [13] and may mediate the increased CVD risk associated with it [14, 15]; the severe insulin-resistant T2D subtype was in fact shown to have the highest risk of developing DKD and coronary artery disease (CAD) [16].

The independent association between IR and risk of death from any cause has been poorly explored in patients with T2D, at variance with those with T1D [17–19]. Moreover, contrasting findings have been reported in nondiabetic individuals, with studies showing either a significant association [20, 21] or no association [22–24] of IR with all-cause mortality.

This study was designed to evaluate the association between IR and death from any cause, beyond traditional CVD risk factors, established CVD, and particularly DKD, in the large cohort of T2D individuals from the Renal Insufficiency And Cardiovascular Events (RIACE) Italian Multicentre Study. Insulin sensitivity was assessed as estimated glucose disposal rate (eGDR), which was originally validated against the euglycaemic-hyperinsulinemic clamp technique [25] and used in epidemiological studies [26, 27] in individuals with T1D. Specifically, we aimed to assess whether eGDR is independently associated with all-cause mortality in T2D individuals or its relationship with death is mediated through the association with DKD.

## Methods

### Design

The RIACE is an observational, prospective, cohort study on the impact of estimated glomerular filtration rate (eGFR) on morbidity and mortality in patients with T2D [28].

### Study population

The RIACE population consists of 15,773 Caucasian individuals with type 2 diabetes (after excluding 160 patients with missing or implausible values), consecutively visiting 19 hospital-based, tertiary referral Diabetes Clinics of the National Health Service throughout Italy in the years 2006–2008. Exclusion criteria were dialysis or renal transplantation.

### All-cause mortality

The vital status of the participants on 31 October 2015 was verified by interrogating the Italian Health Card database (http://sistemats1.sanita.finanze.it/wps/portal/), which provides updated information on all current Italian residents [3].

### Baseline measurements

Baseline data were collected using a standardised protocol across participating centres [28].

Participants underwent a structured interview in order to collect the following information: age, smoking status, known diabetes duration, co-morbidities, and current glucose-, lipid-, and blood pressure (BP)-lowering treatments.

Body mass index (BMI) was calculated from weight and height, whereas waist circumference was estimated from log-transformed BMI values using sex-specific linear regression equations derived from waist measurements obtained from 4618 participants, as previously described [29]. BP was measured with a sphygmomanometer with the patients seated with the arm at the heart level and hypertension was defined as systolic BP > 140 mmHg and/or diastolic BP > 90 and/or anti-hypertensive treatment.

Haemoglobin A_1c_ (HbA_1c_) was measured by high-performance liquid chromatography using DCCT-aligned methods; triglycerides and total and HDL cholesterol were determined in fasting blood samples by colorimetric enzymatic methods; non-HDL cholesterol was calculated by the following formula: total cholesterol − HDL cholesterol; and LDL cholesterol was calculated by the Friedewald formula. Dyslipidaemia was defined as LDL cholesterol > 2.59 mmol/l and/or treatment with lipid-lowering agents.

Presence of DKD was assessed by measuring albuminuria and serum creatinine. As previously detailed [28, 30], albumin excretion rate was obtained from 24-h urine collections or calculated from albumin-to-creatinine ratio in early-morning, first-voided urine samples, using a conversion formula preliminary validated in a subgroup of the RIACE cohort. Albuminuria was measured in fresh urine samples by immunonephelometry or immunoturbidimetry. One-to-three measurements for each patient were obtained; in case of multiple measurements, the geometric mean of 2–3 values was used for analysis. In individuals with multiple measurements, the concordance rate between the first value and the geometric mean was > 90% for all albuminuria categories [30]. Patients were assigned to one of the following categories of albuminuria (mg/24 h): normoalbuminuria (A1, < 30), microalbuminuria (A2, 30–299), or macroalbuminuria (A3, > 300). Serum (and urine) creatinine was measured by the modified Jaffe method, traceable to IDMS, and eGFR was calculated by the CKD Epidemiology Collaboration equation [28]. Patients were assigned to one of the following categories of eGFR (ml·min^−1^·1.73 m^−2^): G1 (> 90), G2 (60–89), G3 (30–59), and G4–5 (< 30). Based on albuminuria and eGFR values, patients were then classified into the following DKD phenotypes [28]: no DKD, albuminuria alone (albuminuric DKD with preserved eGFR), reduced eGFR alone (nonalbuminuric DKD), or albuminuria and reduced eGFR (albuminuric DKD with reduced eGFR).

In each centre, presence of diabetic retinopathy (DR) was evaluated by an expert ophthalmologist by dilated fundoscopy, with grade assigned based on the worst eye [31]. Patients with mild or moderate non-proliferative DR were classified as having non-advanced DR, whereas those with severe non-proliferative DR, proliferative DR, or maculopathy were grouped into the advanced DR category.

Prior major acute CVD events, including myocardial infarction, stroke, foot ulcer/gangrene/amputation, coronary, carotid, and lower limb revascularization, were adjudicated based on hospital discharge records by an ad hoc committee in each centre [32].

As previously described [18], calculation of eGDR was performed according to the following formula: eGDR (mg/kg/min) = 21.158 − (0.09 × waist circumference) − (3.407 × hypertension) − (0.551 × HbA_1c_), where waist circumference is in cm, hypertension is 0 (no) or 1 (yes), and HbA_1c_ is in %. As shown in Additional file 1: Figure S1, the correlation of eGDR calculated using this formula with glucose disposal rate (GDR) measured with euglycaemic-hyperinsulinemic clamp in 140 T2D patients was highly significant (*r* = 0.624; *p* < 0.0001); moreover, it was higher than that of Homeostasis Model Assessment – Insulin resistance (HOMA-IR) in the 85 patients with calculable values (*r* = 0.441; *p* < 0.0001) and similar to that reported in the validation study in T1D individuals [25]. Participants were stratified in tertiles of eGDR calculated using either estimated waist circumference in the whole cohort, i.e. T3 (higher IR), ≤ 4.14; T2, 4.15–5.34; and T1 (lower IR), ≥ 5.35 mg/kg/min, or measured waist circumference in the 4618 individuals with available data, i.e. T3, ≤ 4.11; T2, 4.12–5.54; and T1, ≥ 5.55 mg/kg/min.

### Statistical analysis

Baseline data are expressed as mean ± SD or median (interquartile range) for continuous variables and number of cases (percentage) for categorical variables. Comparisons among eGDR tertiles were performed by one-way ANOVA or Kruskal–Wallis test, according to the parametric or non-parametric distribution of continuous variables, and by Pearson’s *χ*^2^ test for categorical variables.

Binary logistic regression analyses were performed to explore the independent association of tertiles of eGDR (calculated using estimated waist circumference) with albuminuria and eGFR categories or DKD phenotypes at baseline (dependent variables); covariates were age, gender, smoking habits, diabetes duration, dyslipidaemia, non-advanced and advanced DR, prior CVD, cancer, and albuminuria or eGFR (as appropriate according to the dependent variable).

Person-time in years was counted from the index date until the date of death or end of follow-up. Crude mortality rates were reported as events per 1000 person-years (PYs), with 95% Poisson confidence intervals (CIs). Death rates were also adjusted for age and eventually for gender by a Poisson regression model. Kaplan-Meier cumulative survival probabilities for all-cause mortality were estimated according to eGDR tertiles. Differences were analysed with the log-rank statistic. Relative risks according to eGDR tertiles were estimated by Cox proportional hazards regression, adjusted by age and gender (*model 1*), plus albuminuria and eGFR categories (*model 2*) or DKD phenotypes (*model 3*). Furthermore, *model 4* and *model 5* included the variables in *model 2* and *model 3*, respectively, plus multiple confounders excluding variables entering the eGDR formula (waist circumference, HbA_1c_, and hypertension including BP-lowering treatment), i.e. CVD risk factors (smoking habits, diabetes duration, and dyslipidaemia) and complications/comorbidities (DR grade, prior CVD, and cancer). Results are expressed as hazard ratios (HRs) and their 95% CIs. The highest eGDR tertile (T1) was the reference category. Cox proportional hazards regression models were replicated after stratification by age (above and below the median value), gender, prior CVD and DKD phenotypes and appropriate tests were applied for assessing the interaction between each of these variables and the eGDR tertiles. Finally, regression models were rerun using tertiles of eGDR calculated from measured waist circumference.

Tests were 2-sided, and a *p* value < 0.05 was considered statistically significant. Statistical analyses were performed using SPSS version 21.0 (SPSS Inc., Chicago, IL).

## Results

### Baseline clinical features by eGDR tertiles and association of eGDR tertiles with DKD

Baseline characteristics of the study population stratified by eGDR tertiles are shown in Table 1.

**Table 1:** Baseline clinical characteristics of patients with valid information on vital status on October 31, 2015, stratified by eGDR tertiles

Variables	T1 eGDR ≥ 5.35 mg/kg/min	T2 eGDR 4.15–5.34 mg/kg/min	T3 eGDR **<** 4.14 mg/kg/min	***p***
***n*** **(%)**	5218 (33.3)	5219 (33.3)	5219 (33.3)	
**Age, years**	65.5 ± 11.3	68.3 ± 9.6	66.0 ± 9.8	< 0.0001
**Gender,** ***n*** **(%)**				< 0.0001
**Females**	2096 (40.2)	2231 (42.7)	2427 (46.5)	
**Males**	3122 (59.8)	2988 (57.3)	2792 (53.5)	
**Smoking status,** ***n*** **(%)**				< 0.0001
**Never**	2960 (56.7)	2955 (56.6)	2934 (56.2)	
**Former**	1375 (26.4)	1528 (29.3)	1504 (28.8)	
**Current**	883 (16.9)	736 (14.1)	781 (15.0)	
**Diabetes duration, years**	11.6 ± 10.0	13.7 ± 10.5	14.2 ± 9.9	< 0.0001
**HbA**_**1c**_**, mmol/mol**	51.3 ± 13.1	55.6 ± 11.3	70.0 ± 17.8	< 0.0001
**HbA**_**1c**_**, %**	6.84 ± 1.20	7.24 ± 1.03	8.56 ± 1.63	
**Anti-hyperglycaemic treatment,** ***n*** **(%)**				< 0.0001
**Lifestyle**	1029 (19.7)	743 (14.2)	341 (6.5)	
**OHA**	3198 (61.3)	3403 (65.2)	3019 (57.8)	
**OHA + insulin**	314 (6.0)	371 (7.1)	825 (15.8)	
**Insulin**	677 (13.0)	702 (13.5)	1035 (19.8)	
**BMI, kg/m**^**2**^	25.61 ± 3.72	28.06 ± 3.06	33.22 ± 5.10	< 0.0001
**Waist circumference, cm**	95.7 ± 7.6	100.7 ± 6.21	111.1 ± 10.2	< 0.0001
**Triglycerides, mmol/l**	1.17 (0.86, 1.62)	1.32 (0.97, 1.84)	1.54 (1.13, 2.16)	< 0.0001
**Total cholesterol, mmol/l**	4.76 ± 0.96	4.75 ± 0.98	4.81 ± 1.03	0.015
**HDL cholesterol, mmol/l**	1.34 ± 0.37	1.29 ± 0.35	1.23 ± 0.33	< 0.0001
**Non-HDL cholesterol, mmol/l**	3.42 ± 0.91	3.46 ± 0.94	3.58 ± 0.99	< 0.0001
**LDL cholesterol, mmol/l**	2.81 ± 0.82	2.77 ± 0.84	2.77 ± 0.86	0.036
**Lipid-lowering therapy,** ***n*** **(%)**	2013 (38.6)	2607 (50.0)	2618 (50.2)	< 0.0001
**Dyslipidaemia,** ***n*** **(%)**	4090 (78.4)	4395 (84.2)	4371 (83.8)	< 0.0001
**Systolic BP, mmHg**	131.8 ± 16.7	140.6 ± 17.6	141.7 ± 18.2	< 0.0001
**Diastolic BP, mmHg**	76.8 ± 8,7	79.2 ± 9.4	80.3 ± 9.9	< 0.0001
**Pulse pressure, mmHg**	55.1 ± 14.6	61.4 ± 15.8	61.4 ± 15.7	< 0.0001
**Anti-hypertensive therapy,** ***n*** **(%)**	2234 (42.8)	4307 (82.5)	4532 (86.8)	< 0.0001
**RAS blockers,** ***n*** **(%)**	1798 (34.5)	3613 (69.2)	3929 (75.3)	< 0.0001
**Hypertension,** ***n*** **(%)**	2754 (52.8)	5150 (98.7)	5192 (99.5)	< 0.0001
**Anti-platelet therapy,** ***n*** **(%)**	1609 (30.8)	2306 (44.2)	2333 (44.7)	< 0.0001
**Anti-coagulant therapy,** ***n*** **(%)**	152 (2.9)	243 (4.7)	274 (5.3)	< 0.0001
**DR,** ***n*** **(%)**				< 0.0001
**No**	4396 (84.2)	4128 (79.1)	3665 (70.2)	
**Non-advanced**	484 (9.3)	624 (11.9)	839 (16.1)	
**Advanced**	338 (6.5)	467 (8.9)	715 (13.7)	
**Albuminuria, mg/24 h**	11.2 (5.8, 22.2)	13.1 (6.5, 32.5)	17.1 (8.1, 52.6)	< 0.0001
**Albuminuria categories**				< 0.0001
**A1 (normoalbuminuria)**	4226 (81.0)	3842 (73.6)	3392 (65.0)	
**A2 (microalbuminuria)**	861 (16.5)	1160 (22.2)	1444 (27.7)	
**A3 (macroalbuminuria)**	131 (2.5)	217 (4.2)	383 (7.3)	
**Serum creatinine, μmol/l**	70.8 ± 15.7	74.0 ± 17.0	114.2 ± 35.1	< 0.0001
**eGFR, ml·min**^**−1**^**·1.73 m**^**−2**^	83.8 ± 20.1	78.3 ± 20.3	78.8 ± 22.0	< 0.0001
**eGFR categories**				< 0.0001
**G1 (>** **90 ml·min**^**−1**^**·1.73 m**^**−2**^**)**	2253 (43.2)	1691 (32.4)	1832 (35.1)	
**G2 (60–89 ml·min**^**−1**^**·1.73 m**^**−2**^**)**	2303 (44.1)	2533 (48.5)	2338 (44.8)	
**G3 (30–59 ml·min**^**−1**^**·1.73 m**^**−2**^**)**	593 (11.4)	906 (17.4)	928 (17.8)	
**G4–5 (< 30 ml·min**^**−1**^**·1.73 m**^**−2**^**)**	69 (1.3)	89 (1.7)	121 (2.3)	
**DKD phenotypes**				< 0.0001
**No DKD**	3837 (73.5)	3294 (63.1)	2853 (54,7)	
**Albuminuric DKD with preserved eGFR**	719 (13.8)	930 (17.8)	1317 (25.2)	
**Nonalbuminuric DKD**	389 (7.5)	548 (10.5)	539 (10.3)	
**Albuminuric DKD with reduced eGFR**	273 (5.2)	447 (8.6)	510 (9.8)	
**CVD,** ***n*** **(%)**				
**Any**	889 (17.0)	1324 (25.4)	1407 (26.9)	< 0.0001
**Acute myocardial infarction**	412 (7.9)	652 (12.5)	678 (13.0)	< 0.0001
**Coronary revascularization**	361 (6.9)	578 (11.1)	640 (12.3)	< 0.0001
**Any coronary event**	558 (10.7)	892 (17.1)	946 (18.1)	< 0.0001
**Stroke**	134 (2.6)	190 (3.6)	189 (3.6)	0.002
**Carotid revascularization**	191 (3.7)	303 (5.8)	362 (6.9)	< 0.0001
**Any cerebro-vascular event**	307 (5.9)	467 (8.9)	518 (9.9)	< 0.0001
**Ulcer/gangrene/amputation**	144 (2.8)	183 (3.5)	229 (4.4)	< 0.0001
**Lower limb revascularization**	103 (2.0)	171 (3.3)	176 (3.4)	< 0.0001
**Any peripheral event**	214 (4.1)	309 (5.9)	360 (6.9)	< 0.0001
**Cancer,** ***n*** **(%)**	358 (6.9)	359 (6.9)	314 (6.0)	0.127

Values are mean ± SD or median (interquartile range) for continuous variables and number of cases (percentage) for categorical variables. *eGDR* estimated glucose disposal rate, *T1* tertile 1, higher insulin sensitivity, *T2* tertile 2, *T3* tertile 3, higher insulin resistance, *HbA*_*1c*_ haemoglobinA_1c_, *OHA* oral hypoglycaemic agents, *BMI* body mass index, *BP* blood pressure, *RAS* renin-angiotensin system, *DR* diabetic retinopathy, *eGFR* estimated glomerular filtration rate, *DKD* diabetic kidney disease, *CVD* cardiovascular disease

Compared with participants in the T1 group (reference), those in the T2 and T3 groups were more frequently females and former smokers, had longer diabetes duration, higher BMI, triglycerides, and non-HDL cholesterol, and lower HDL cholesterol, with no meaningful difference in total and LDL cholesterol. As expected, T2 and T3 participants had higher HbA_1c_, waist circumference, BP levels, and prevalence of hypertension, dyslipidaemia, and treatment with insulin, alone or combined with non-insulin agents, anti-hypertensive drugs, RAS blockers, and lipid-lowering agents.

Levels of albuminuria and prevalence of micro and macroalbuminuria and of the albuminuric DKD phenotypes increased progressively from T1 to T3, whereas eGFR decreased and prevalence of nonalbuminuric DKD increased from T1 to T2, with no further change in T3. Prevalence of non-advanced and advanced DR and prior CVD, either as a whole or by vascular bed, increased from T1 to T3, whereas no difference was observed in cancer prevalence at baseline among eGDR tertiles.

Binary logistic regression analyses exploring the relationship between eGDR tertiles and DKD showed an independent association with micro- and macroalbuminuria and the albuminuric DKD phenotypes (albuminuria alone or combined with reduced eGFR) as well as with eGFR categories and the nonalbuminuric DKD phenotype (Additional file 2: Table S1).

### Association of eGDR tertiles with all-cause mortality

**Fig. 1:**
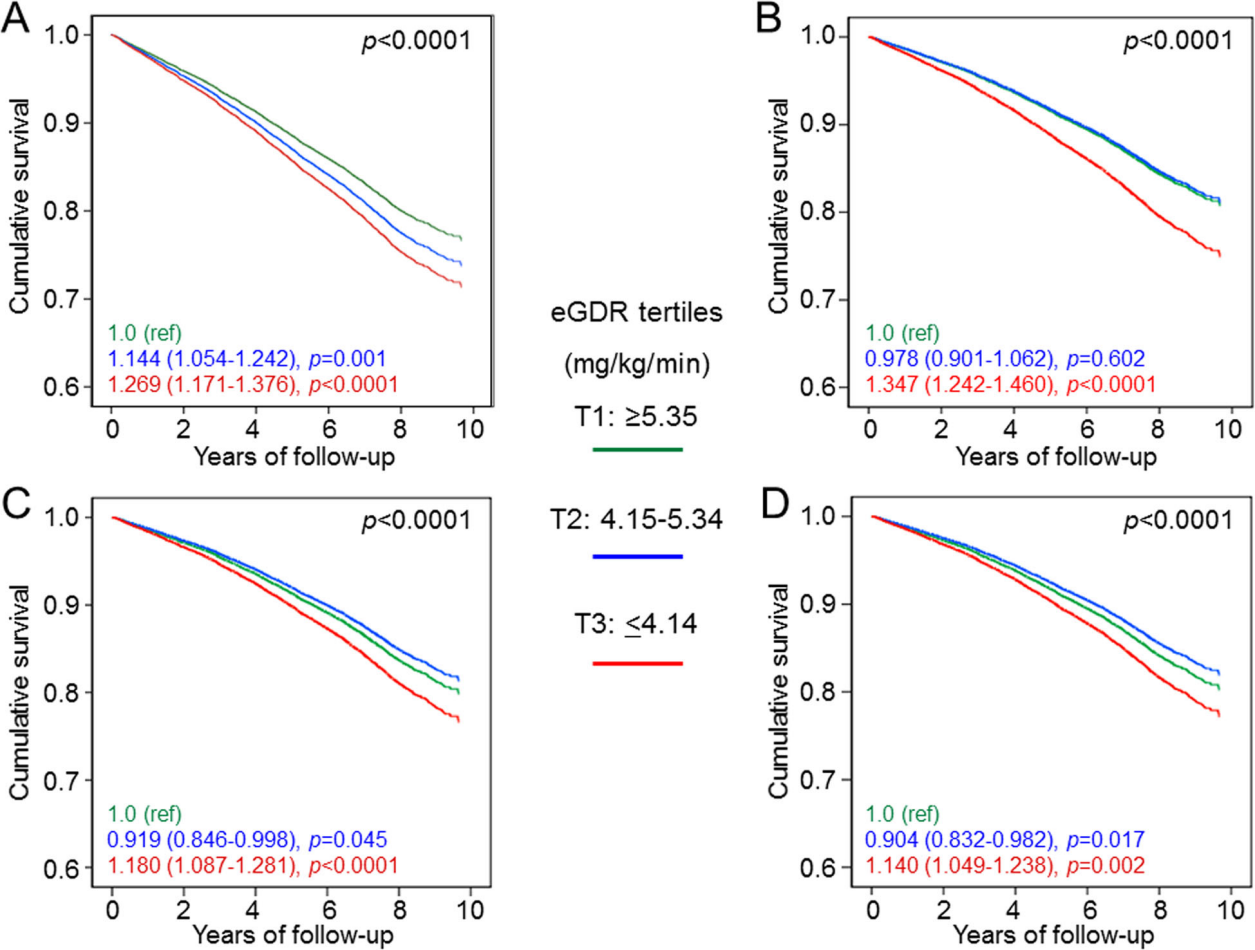
Cox proportional hazards regression, unadjusted (**a**), and adjusted by age and gender (**b**; model 1), plus albuminuria and eGFR categories (**c**; model 2) plus multiple confounders (**d**; model 4), according to eGDR tertiles. HRs (95% CI) for mortality are shown for each tertile. CVD risk factors (smoking habits, diabetes duration, and dyslipidaemia) and complications/comorbidities (DR grade, prior CVD, and cancer). HR, hazard ratio; CI, confidence interval; eGFR, estimated glomerular filtration rate; eGDR, estimated glucose disposal rate; HR, hazard ratio; CI, confidence interval; CVD, cardiovascular disease; DR, diabetic retinopathy

The vital status on October 31, 2015, was retrieved for 15,656 participants (99.3% of the original cohort). A total of 3602 deaths occurred during the 116,100 PYs of follow-up (23.01%; 31.02 per 1000 PYs [95% CI 30.01–32.04]) over a mean observation of 7.42 ± 2.05 years [3]. Death rates (Table 2), Kaplan-Meier estimates (Additional file 3: Figure S2) and unadjusted HRs (Fig. 1a) increased progressively across eGDR categories; however, the age-adjusted death rates for T2 and T1 were superimposable (Table 2), as those adjusted for age and gender (16.40 vs 16.74 per 1000 PYs, *p* = 0.620), with a significantly increased death rate vs T1 only for T3, i.e. for the subgroup with the lowest insulin sensitivity (22.35 per 1000 PYs when adjusting for age and gender, *p* < 0.0001).

**Table 2:** Mortality rates according to eGDR tertiles in the whole population, unadjusted and age-adjusted

	***N***	Events	Percent events	Events per 1000 patient-years (95% CI) unadjusted	***p***	Events per 1000 patient-years (95% CI) age-adjusted	***p***
**eGDR tertiles**					< 0.0001		< 0.0001
**T1**	5218	1075	20.60	27.35 (25.71–28.98)	Ref	21.71 (20.33–23.17)	Ref
**T2**	5219	1207	23.13	31.23 (29.47–33,00)	0.002*	21.19 (19.89–22.57)	0.565*
**T3**	5219	1320	25.29	34.60 (32.73–36.47)	< 0.0001* 0.010**	28.15 (26.56–29.83)	< 0.0001* < 0.0001**

*eGDR* estimated glucose disposal rate, *CI* confidence interval, *K-M* Kaplan-Meier. *p value vs T1; **p value vs T2

At Cox regression adjusted for age and gender (Fig. 1b, Additional file 4: Table S2*, model 1*) and for age, gender, and albuminuria and eGFR categories (Fig. 1c, Additional file 4: Table S2*, model 2*), the HRs vs T1 for all-cause mortality were increased for T3, but not for T2. Similar results were obtained by adjusting for DKD phenotypes in addition to age and gender (Additional file 4: Table S2, *model 3*). Further adjusting for several confounders, HRs for all-cause mortality remained higher for T3 vs T1 in either the model including albuminuria and eGFR categories (Fig. 1d, Additional file 5: Table S3, *model 4*) or that including DKD phenotypes (Additional file 5: Table S3, *model 5*).

The results were similar when using tertiles of eGDR calculated from measured waist circumference in the 4618 individuals with available data (Additional file 6: Figure S3).

### Association of eGDR tertiles with all-cause mortality by age, gender and prior CVD

Crude and age-adjusted death rates were increased for T3 vs T1 in individuals below and above median age and in both males and females, but only in participants without history of CVD (Table 3), whereas adjusted HRs were increased in both age groups (though more in younger than in older individuals), but only in males and participants with prior CVD, though interaction was significant only for age (Additional file 7: Table S4).

**Table 3:** Mortality rates and Kaplan-Meier estimates according to eGDR tertiles in subgroups, unadjusted and age-adjusted

	***N***	Events	Percent events	Events per 1000 patient-years (95% CI) unadjusted	***p***	Events per 1000 patient-years (95% CI) age-adjusted	***p***
**Age**							
**Below median value (*n*** **= 7829)**					< 0.0001		< 0.0001
**T1**	2803	238	8.5	10.52 (9.26–11.94)	Ref	16.29 (14.01–18.94)	Ref
**T2**	2284	218	9.5	11.94 (10.45–13.63)	0.179 *	16.59 (14.33–19.21)	0.845*
**T3**	2742	403	14.7	18.92 (17.16–20.86)	< 0.0001 * < 0.0001 **	27.76 (24.57–31.36)	< 0.0001* < 0.0001**
K-M log rank 63.68; *p* < 0.0001							
**Above median value (*n*** **= 7827)**					0.042		< 0.0001
**T1**	2415	837	34.7	50.20 (46.91–53.72)	Ref	19.08 (17.29–21.05)	Ref
**T2**	2935	989	33,7	48.53 (45.60–51.65)	0.473 *	18.66 (17.01–20.48)	0.642*
**T3**	2477	917	37.0	54.42 (51.01–58.06)	0.091 * 0.013 **	23.01 (21.04–25.16)	< 0.0001* < 0.0001**
K-M log rank 7.02; *p* = 0.030							
**Gender**							
**Males (*n*** **= 8902)**					< 0.0001		< 0.0001
**T1**	3122	686	22.0	29.34 (27.23–31.62)	Ref	25.48 (23.53–27.60)	Ref
**T2**	2988	736	24.6	33.50 (31.17–36.01)	0.013*	24.38 (22.51–26.40)	0.404*
**T3**	2792	765	27.4	38.10 (35.49–40.90)	< 0.0001* 0.013**	34.15 (31.69–36.80)	< 0.0001* < 0.0001**
K-M log rank 25.25; *p* < 0.0001							
**Females (*n*** **= 6754)**					0.002		< 0.0001
**T1**	2096	389	18.6	24.42 (22.11–26.97)	Ref	16.21 (14.46–18.17)	Ref
**T2**	2231	471	21.1	28.24 (25.80–30.91)	0.033*	16.54 (14.88–18.39)	0.764*
**T3**	2427	555	22.9	30.72 (28.26–33.38)	0.180* < 0.0001**	21.65 (19.71–23.79)	< 0.0001* < 0.0001**
K-M log rank 12.55; *p* = 0.002							
**Prior CVD**							
**No (*n*** **= 12,036)**					< 0.0001		< 0.0001
**T1**	4329	728	16.8	21.80 (20.27–23.44)	Ref	18.38 (16.99–19.89)	Ref
**T2**	3895	732	18.8	24.72 (22.99–26.57)	0.017*	17.60 (16.25–19.06)	0.403*
**T3**	3812	773	20.3	26.89 (25.06–28.85)	< 0.0001* 0.103**	22.43 (20.80–24.18)	< 0.0001* < 0.0001**
K-M Log rank 17.16; *p* < 0.0001							
**Yes (*n*** **= 3620)**					0.174		< 0.0001
**T1**	889	347	39.0	58.74 (52.88–65.26)	Ref	37.96 (33.69–42.78)	Ref
**T2**	1324	475	35.9	52.61 (48.08–57.56)	0.122*	33.81 (30.42–37.58)	0.105*
**T3**	1407	547	38.9	58.19 (53.51–63.28)	0.890* 0.107**	46.58 (42.51–51.05)	0.003* < 0.0001**
K-M log rank 3.60; *p* = 0.165							

*eGDR* estimated glucose disposal rate, *CI* confidence interval, *CVD* cardiovascular disease, *K-M* Kaplan-Meier. *p value vs T1; **p value vs T2

### Association of eGDR tertiles with all-cause mortality by DKD phenotypes

**Fig. 2:**
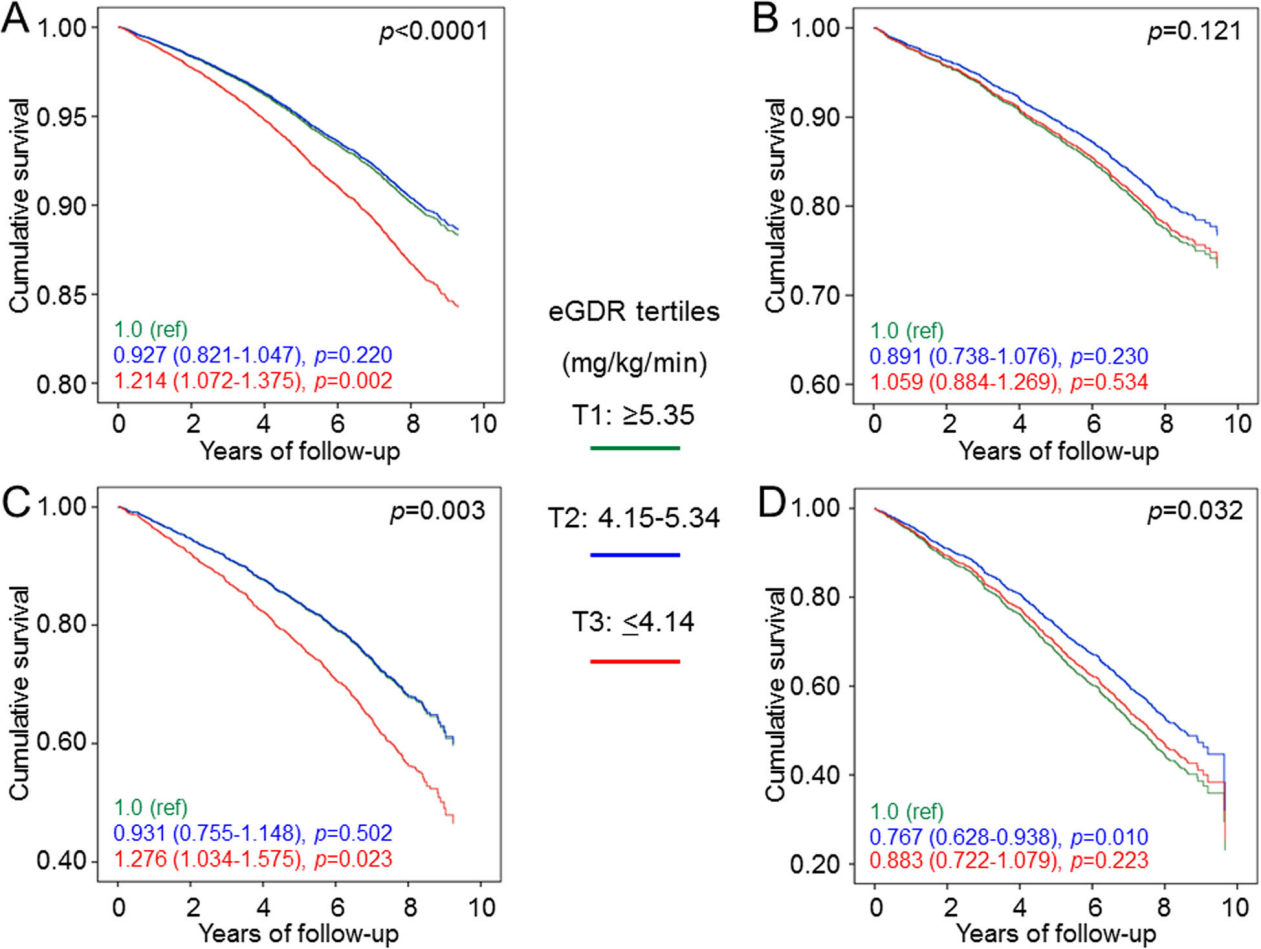
Cox proportional hazards regression, adjusted by age and gender, albuminuria and eGFR categories, and multiple confounders, according to eGDR tertiles, in participants with no DKD (**a**), albuminuric DKD with preserved eGFR (**b**), nonalbuminuric DKD (**c**) and albuminuric DKD with reduced eGFR (**d**). HRs (95% CI) for mortality are shown for each tertile. CVD risk factors (smoking habits, diabetes duration, and dyslipidaemia) and complications/comorbidities (DR grade, prior CVD, and cancer). eGFR, estimated glomerular filtration rate; eGDR, estimated glucose disposal rate; DKD, diabetic kidney disease; HR, hazard ratio; CI, confidence interval; CVD, cardiovascular disease; DR, diabetic retinopathy

After stratification by DKD phenotypes, age-adjusted death rates (Table 4) and adjusted HRs (Fig. 2) were higher for T3 vs T1 in individuals with no DKD and or nonalbuminuric DKD, but not in those with albuminuric DKD with preserved or reduced eGFR. The interaction between eGDR and DKD phenotypes was significant (*p* = 0.018).

**Table 4:** Mortality rates and Kaplan-Meier estimates according to eGDR tertiles by DKD phenotypes, unadjusted and age-adjusted

	***N***	Events	Percent events	Events per 1000 patient-years (95% CI) unadjusted	***p***	Events per 1000 patient-years (95% CI) age-adjusted	***p***
**No DKD (*n*** **= 9984)**					0.023		< 0.0001
**T1**	3837	553	14.4	18.44 (16.97–20.04)	Ref	17.24 (15.79–18.83)	Ref
**T2**	3294	504	14.3	19.78 (18.12–21.58)	0.257*	15.81 (14.41–17.36)	0.160*
**T3**	2853	479	16.8	21.93 (20.06–23.99)	0.006* 0.106**	20.47 (18.66–22.46)	0.007* < 0.0001**
K-M log rank 7.94; *p* = 0.019							
**Albuminuric DKD with preserved eGFR (*n*** **= 2966)**					0.639		0.101
**T1**	719	199	27.7	38.48 (33.49–44.21)	Ref	30.89 (26.61–35.84)	Ref
**T2**	930	251	27.0	37.35 (33.00–42.27)	0.754*	29.42 (25.76–33.60)	0.610*
**T3**	1317	343	26.0	35.49 (31.93–39.46)	0.371* 0.542**	34.97 (31.37–38.99)	0.161* 0.037**
K-M log rank 0.93; *p* = 0.629							
**Nonalbuminuric DKD (*n*** **= 1476)**					0.424		0.005
**T1**	389	155	39.8	59.36 (50.71–69,48)	Ref	27.48 (22.28–33.89)	Ref
**T2**	548	217	39.6	58.77 (51.44–67.13)	0.924*	26.59 (21.99–32.14)	0.754*
**T3**	539	232	43.0	65.94 (57.98–74.99)	0.307* 0.223**	35.78 (30.16–42.45)	0.010* 0.002**
K-M log rang 1.990; *p* = 0.370							
**Albuminuric DKD with reduced eGFR (*n*** **= 1230)**					0.034		0.074
**T1**	273	168	61.5	109.36 (94.01–127.21)	Ref	67.64 (56.36–81.17)	Ref
**T2**	447	235	52.6	85.54 (75.27–97.20)	0.019*	53.62 (45.71–62.89)	0.026*
**T3**	510	266	52.2	85.00 (75.37–95.85)	0.014* 0.944**	60.21 (52.27–69.36)	0.251* 0.197**
K-M log rank 8.78; *p* = 0.012							

*DKD* diabetic kidney disease, *eGDR* estimated glucose disposal rate, *CI* confidence interval, *K-M* Kaplan-Meier. *p value vs T1; **p value vs T2

## Discussion

This analysis of the RIACE cohort of individuals with T2D showed a significant association between IR, as assessed by eGDR, and all-cause mortality. This relationship was independent of traditional CVD risk factors clustering with impaired insulin sensitivity as well as of cardiorenal complications and cancer, the risk of which has also been associated with IR. Regarding DKD, which was the main focus of our study, adjustment for albuminuria and eGFR or DKD phenotypes attenuated only slightly the association between eGDR and mortality, consistent with a previous report in T1D patients from the Swedish National Diabetes Register [18]. However, two other studies in T1D individuals showed no significant association with all-cause mortality when serum creatinine [17] and albuminuria and eGFR or DKD [19] were included in the regression models. Likewise, in older adults without diabetes, the association of the insulin sensitivity index or fasting insulin concentration with all-cause mortality disappeared after adjustment for eGFR [22]. Moreover, when adjusting for confounders including eGFR, glucose disposal rate measured by the euglycaemic hyperinsulinemic clamp technique was no longer associated with all-cause mortality in patients with chronic kidney disease (CKD) stages 3 and 4 [33] and HOMA-IR was not an independent predictor of death in individuals with mild-to-moderate CKD from the Chronic Renal Insufficiency Cohort Study [34]. Indeed, in our study, eGDR was independently associated with micro- and macroalbuminuria and the albuminuric DKD phenotypes, consistent with previous reports [35, 36], as well as with eGFR categories or the nonalbuminuric DKD phenotype. However, eGDR was independently associated with all-cause death in individuals with no DKD or the nonalbuminuric DKD phenotype, but not in patients with albuminuria with preserved or reduced eGFR, suggesting that, in these individuals, the impact of IR on mortality is mediated by albuminuria.

Taken together, these findings indicate that the impact of IR on mortality in T2D individuals is only partly mediated by the increased prevalence of CVD risk factors and complications/comorbidities, including DKD. This may imply either that IR exerts direct deleterious effects on survival or that the increased risk of death is explained by unmeasured confounders associated with IR or by the inability of “statistical” adjustment to fully account for the impact of measured confounders. Low-grade chronic inflammation, which is also associated with IR but was not accounted for in the regression models, may have played a role in favouring the increased mortality observed in the lowest eGDR tertile (T3). This interpretation is consistent with a previous study in diabetic patients showing that C-reactive protein (CRP) was an independent predictor of mortality in addition to IR [37]. Moreover, the combination of IR, as assessed by HOMA-IR, and systemic inflammation, as assessed by CRP, was associated with all-cause and CVD mortality in community-dwelling older individuals from the InCHIANTI Study [38], whereas Lee et al. showed that CRP was an independent predictor of all-cause and cancer-related, but not CVD mortality, irrespective of HOMA-IR [39]. Finally, CRP was included among the covariates that masked the association of the insulin sensitivity index or fasting insulin concentration with all-cause mortality in older adults without diabetes [22].

To the best of our knowledge, this is the first study exploring the independent association of IR with mortality in a large sample of unselected patients with T2D. In fact, one study has previously assessed the ability of eGDR, as a measure of IR, to predict mortality in individuals with T2D and CAD who underwent coronary artery bypass grafting. This nationwide, population-based cohort study reported a significant independent association of the lowest vs the highest eGDR tertile with all-cause death [adjusted HR, 1.46 (95% CI, 1.12–1.90)] and a composite of major adverse CVD events and all-cause death (adjusted HR, 1.29 [95% CI 1.04–1.60]) over a 3.1-year follow-up in 3256 T2D patients [40]. Consistently, another study in 350 Japanese patients with T2D reported that lower insulin sensitivity, measured as K index of the insulin tolerance test, was an independent predictor of all-cause mortality and CVD events [37].

Conversely, more robust findings were reported in T1D, where eGDR was originally developed. An independent association was in fact shown with all-cause mortality [17] and CAD [26] over a 10-year follow-up in the Pittsburgh Epidemiology of Diabetes Complications Study and with all-cause mortality, CVD morbidity and mortality, and the combined end-point of CVD events and death over a 7.1-year follow-up in 17,050 individuals from the Swedish National Diabetes Register [18]. Recently, a single-centre, 10-year observational study confirmed that eGDR was an independent predictor of major CVD events, CAD, and all-cause mortality in T1D patients [19].

In our study, risk of death increased stepwise in T2 and T3 vs T1 (by 14% and 27%, respectively). However, after adjustment for age and gender, mortality risk was similar in T2 and T1, whereas it was 35% higher in T3 vs T1. Moreover, risk of death remained significantly higher in T3 vs T1 after further adjustment for albuminuria and eGFR or, alternatively, for DKD phenotypes (by approximately 20%) and even when other CVD risk factors, DR, CVD and cancer were included in the models (by 14%). Thus, in patients with T2D, the association between eGDR and all-cause death, thought significant, was less strong than that reported in patients with T1D. In fact, in T1D individuals, mortality risk was about 2.2-fold in those with the lowest compared to those with the highest eGDR values [18] and was 40–50% lower for each SD increase in eGDR [17, 19]. These findings are consistent with the different weight of confounders such as traditional CVD risk factors and complications/comorbidities in the two clinical settings.

To further explore the role of these confounders, we conducted subgroup analyses by gender, age, prior CVD, and DKD phenotypes. The finding that the association between eGDR and mortality was stronger in younger individuals and in those with no prior CVD (and DKD) is in keeping with the concept that the impact of IR per se on mortality risk is higher in individuals at lower risk, such as those with T1D. This interpretation is consistent with previous studies in nondiabetic individuals from the third National Health and Nutrition Examination Survey showing an independent association of IR, as assessed by HOMA-IR, with all-cause mortality only in those with normal BMI [20].

Strengths of this study are the large sample size, the long observation period, the completeness of data collected at baseline and follow-up, the wide range of clinical parameters assessed, and the accurate determination of mortality due to the high quality of the Italian Health Card Database. There are also several limitations. First, as for other surrogate measures of IR, eGDR may not be as accurate as GDR assessed by the euglycaemic hyperinsulinemic clamp technique, which however is not applicable to large cohorts. Second, eGDR was validated against the euglycaemic hyperinsulinemic clamp technique in patients with T1D [25], though it was used also in those with T2D for assessing the relationship between IR and mortality [40]. However, indices which require measurement of insulin (or C-peptide) levels, such as HOMA-IR, are not suitable for estimating insulin sensitivity in an unselected population of individuals with type 2 diabetes including a large proportion (~ 25) of insulin-treated patients such as the RIACE cohort. Moreover, we showed that eGDR correlated significantly (and better than HOMA-IR) with clamp-derived GDR data from T2D individuals. Third, eGDR was calculated using waist circumference estimated from BMI, but results did not change when repeating the analyses with eGDR calculated using measured waist circumference values, even in the smaller sample of 4618 individuals with available data. Fourth, the observational design makes causal interpretation impossible and does not allow to rule out the effect of unmeasured confounders, such as inflammatory markers. Fifth, the study findings may not be applicable to the general ambulatory population, as only part of the individuals with type 2 diabetes attend Diabetes Clinics in Italy. Finally, potential limitations concerning non-centralization of assessments of CVD risk factors and complications have been extensively addressed elsewhere [3, 28–32].

## Conclusions

This study investigated the ability of IR, as assessed as eGDR, in predicting mortality in a large population of unselected T2D individuals. Participants in the lowest eGDR tertile (highest IR) had the worst CVD risk profile and the highest prevalence of DKD, DR, and prior CVD events, as compared with those in the highest eGDR tertile. After adjustment for all these confounders, including DKD, risk of death remained significantly associated with mortality, suggesting that IR is an independent predictor of death from any cause in T2D individuals. However, eGDR was independently associated with all-cause death in patients with no DKD and nonalbuminuric DKD, but not in those with the albuminuric phenotypes, suggesting that in these individuals the effect of eGDR was mediated by its strong independent association with albuminuria. Moreover, the impact of IR was stronger in males and in younger participants and was significant only in those without CVD. Our findings suggest including measures of IR to improve risk stratification for preventive and therapeutic purposes in individuals with T2D.

## Supplementary Information

**Additional file 1: Figure S1.** Scatterplot of clamp-derived GDR and eGDR (A) or HOMA-IR (B) in 140 individuals with T2D.

**Additional file 2: Table S1.** Binary logistic regression analyses of kidney parameters (albuminuria categories, eGFR categories, and DKD phenotypes as dependent variables) with eGDR tertiles as covariate adjusted for confounders*.

**Additional file 3: Figure S2.** Cumulative survival by Kaplan Meier analysis according to eGDR tertiles. Numbers (percentages) of death are shown for each tertile.

**Additional file 4: Table S2.** Survival analysis by Cox proportional hazards regression according to eGDR tertiles, adjusted for age and gender (*Model 1*) and for age and gender plus albuminuria and eGFR categories (*Model 2*) or DKD phenotypes (*Model 3*).

**Additional file 5: Table S3.** Survival analysis by Cox proportional hazards regression according to eGDR tertiles, adjusted for age, gender, albuminuria and eGFR categories (*Model 4*) or DKD phenotypes (*Model 5*), plus multiple confounders*.

**Additional file 6: Figure S3.** Cox proportional hazards regression, unadjusted (A), and adjusted by age and gender (B; model 1), plus albuminuria and eGFR categories (C; model 2) plus multiple confounders* (D; model 4), according to tertiles of eGDR calculated using measured waist circumference. HRs (95% CI) for mortality are shown for each tertile.

**Additional file 7: Table S4.** Survival analysis by Cox proportional hazards regression according to eGDR tertiles in subgroups.

## Data Availability

The datasets analysed during the current study are available from the corresponding author on reasonable request.

## Abbreviations

BMI: Body mass index
BP: Blood pressure
CI: Confidence interval
CKD: Chronic kidney disease
CVD: Cardiovascular disease
DKD: Diabetic kidney disease
DR: Diabetic retinopathy
eGDR: Estimated glucose disposal rate
eGFR: Estimated glomerular filtration rate
HbA_1c_: Haemoglobin A_1c_
GDR: Glucose disposal rate
HOMA-IR: Homeostasis Model Assessment – Insulin Resistance
HR: Hazard ratio
KDIGO: Kidney Disease: Improving Global Outcomes
IR: Insulin resistance
PYs: Person-years
RAS: Renin-angiotensin system
RIACE: Renal Insufficiency And Cardiovascular Events
T1D: Type 1 diabetes
T2D: Type 2 diabetes

## References

[1] Seshasai SR, Kaptoge S, Thompson A, Di Angelantonio E, Gao P, Emerging Risk Factors Collaboration (2011). Diabetes mellitus, fasting glucose, and risk of cause-specific death. N Engl J Med.

[2] Tancredi M, Rosengren A, Svensson AM, Kosiborod M, Pivodic A, Gudbjörnsdottir S (2015). Excess mortality among persons with type 2 diabetes. N Engl J Med.

[3] Penno G, Solini A, Bonora E, Orsi E, Fondelli C, Zerbini G (2018). Defining the contribution of chronic kidney disease to all-cause mortality in patients with type 2 diabetes: the Renal Insufficiency And Cardiovascular Events (RIACE) Italian Multicenter Study. Acta Diabetol.

[4] Rawshani A, Rawshani A, Franzén S, Eliasson B, Svensson AM, Miftaraj M (2017). Mortality and cardiovascular disease in type 1 and type 2 diabetes. N Engl J Med.

[5] Gregg EW, Cheng YJ, Srinivasan M, Lin J, Geiss LS, Albright AL (2018). Trends in cause-specific mortality among adults with and without diagnosed diabetes in the USA: an epidemiological analysis of linked national survey and vital statistics data. Lancet..

[6] Gæde P, Oellgaard J, Carstensen B, Rossing P, Lund-Andersen H, Parving HH (2016). Years of life gained by multifactorial intervention in patients with type 2 diabetes mellitus and microalbuminuria: 21 years follow-up on the Steno-2 randomised trial. Diabetologia..

[7] Oellgaard J, Gæde P, Rossing P, Persson F, Parving HH, Pedersen O (2017). Intensified multifactorial intervention in type 2 diabetics with microalbuminuria leads to long-term renal benefits. Kidney Int.

[8] Patel TP, Rawal K, Bagchi AK, Akolkar G, Bernardes N, Dias DDS (2016). Insulin resistance: an additional risk factor in the pathogenesis of cardiovascular disease in type 2 diabetes. Heart Fail Rev.

[9] Laakso M, Kuusisto J (2014). Insulin resistance and hyperglycaemia in cardiovascular disease development. Nat Rev Endocrinol.

[10] Kilpatrick ES, Rigby AS, Atkin SL (2007). Insulin resistance, the metabolic syndrome, and complication risk in type 1 diabetes: “double diabetes” in the diabetes control and complications trial. Diabetes Care.

[11] van Sloten TT, Henry RM, Dekker JM, Nijpels G, Unger T, Schram MT (2014). Endothelial dysfunction plays a key role in increasing cardiovascular risk in type 2 diabetes: the Hoorn study. Hypertension..

[12] Sakkinen PA, Wahl P, Cushman M, Lewis MR, Tracy RP (2000). Clustering of procoagulation, inflammation, and fibrinolysis variables with metabolic factors in insulin resistance syndrome. Am J Epidemiol.

[13] Karalliedde J, Gnudi L (2016). Diabetes mellitus, a complex and heterogeneous disease, and the role of insulin resistance as a determinant of diabetic kidney disease. Nephrol Dial Transplant.

[14] Spoto B, Pisano A, Zoccali C (2016). Insulin resistance in chronic kidney disease: a systematic review. Am J Physiol Renal Physiol.

[15] Leyking S, Fliser D (2014). Insulin resistance in CKD. Clin J Am Soc Nephrol.

[16] Ahlqvist E, Storm P, Käräjämäki A, Martinell M, Dorkhan M, Carlsson A (2018). Novel subgroups of adult-onset diabetes and their association with outcomes: a data-driven cluster analysis of six variables. Lancet Diabetes Endocrinol.

[17] Olson JC, Erbey JR, Williams KV, Becker DJ, Edmundowicz D, Kelsey SF (2002). Subclinical atherosclerosis and estimated glucose disposal rate as predictors of mortality in type 1 diabetes. Ann Epidemiol.

[18] Nyström T, Holzmann MJ, Eliasson B, Svensson AM, Sartipy U (2018). Estimated glucose disposal rate predicts mortality in adults with type 1 diabetes. Diabetes Obes Metab.

[19] Garofolo M, Gualdani E, Scarale MG, Bianchi C, Aragona M, Campi F (2020). Insulin resistance and risk of major vascular events and all-cause mortality in type 1 diabetes: a 10-year follow-up study. Diabetes Care.

[20] Ausk KJ, Boyko EJ, Ioannou GN (2010). Insulin resistance predicts mortality in nondiabetic individuals in the U.S. Diabetes Care.

[21] Pan K, Chlebowski RT, Mortimer JE, Gunther MJ, Rohan T, Vitolins MZ (2020). Insulin resistance and breast cancer incidence and mortality in postmenopausal women in the Women's Health Initiative. Cancer..

[22] de Boer IH, Katz R, Chonchol MB, Fried LF, Ix JH, Kestenbaum B (2012). Insulin resistance, cystatin C, and mortality among older adults. Diabetes Care.

[23] Barr EL, Cameron AJ, Balkau B, Zimmet PZ, Welborn TA, Tonkin AM (2010). HOMA insulin sensitivity index and the risk of all-cause mortality and cardiovascular disease events in the general population: the Australian Diabetes, Obesity and Lifestyle Study (AusDiab) study. Diabetologia..

[24] Welsh P, Preiss D, Lloyd SM, de Craen AJ, Jukema JW, Westendorp RG (2014). Contrasting associations of insulin resistance with diabetes, cardiovascular disease and all-cause mortality in the elderly: PROSPER long-term follow-up. Diabetologia..

[25] Williams KV, Erbey JR, Becker D, Arslanian S, Orchard TJ (2000). Can clinical factors estimate insulin resistance in type 1 diabetes?. Diabetes..

[26] Orchard TJ, Olson JC, Erbey JR, Williams K, Forrest KY, Smithline Kinder L (2003). Insulin resistance-related factors, but not glycemia, predict coronary artery disease in type 1 diabetes: 10-year follow-up data from the Pittsburgh Epidemiology of Diabetes Complications Study. Diabetes Care.

[27] Epstein EJ, Osman JL, Cohen HW, Rajpathak SN, Lewis O, Crandall JP (2013). Use of the estimated glucose disposal rate as a measure of insulin resistance in an urban multiethnic population with type 1 diabetes. Diabetes Care.

[28] Penno G, Solini A, Bonora E, Fondelli C, Orsi E, Zerbini G, Trevisan R (2011). Clinical significance of nonalbuminuric renal impairment in type 2 diabetes. J Hypertens.

[29] Penno G, Solini A, Bonora E, Fondelli C, Orsi E, Zerbini G, Trevisan R (2013). Gender differences in cardiovascular disease risk factors, treatments and complications in patients with type 2 diabetes: the RIACE Italian multicentre study. J Intern Med.

[30] Pugliese G, Solini A, Fondelli C, Trevisan R, Vedovato M, Nicolucci A (2011). Reproducibility of albuminuria in type 2 diabetic subjects. Findings from the Renal Insufficiency And Cardiovascular Events (RIACE) study. Nephrol Dial Transplant.

[31] Penno G, Solini A, Zoppini G, Orsi E, Zerbini G, Trevisan R (2012). Rate and determinants of association between advanced retinopathy and chronic kidney disease in patients with type 2 diabetes: the Renal Insufficiency And Cardiovascular Events (RIACE) Italian multicenter study. Diabetes Care.

[32] Solini A, Penno G, Bonora E, Fondelli C, Orsi E, Arosio M (2012). Diverging association of reduced glomerular filtration rate and albuminuria with coronary and noncoronary events in patients with type 2 diabetes: the renal insufficiency and cardiovascular events (RIACE) Italian multicenter study. Diabetes Care.

[33] Xu H, Huang X, Arnlov J, Cederholm T, Stenvinkel P, Lindholm B (2014). Clinical correlates of insulin sensitivity and its association with mortality among men with CKD stages 3 and 4. Clin J Am Soc Nephrol.

[34] Schrauben SJ, Jepson C, Hsu JY, Wilson FP, Zhang X, Lash JP (2019). Insulin resistance and chronic kidney disease progression, cardiovascular events, and death: findings from the chronic renal insufficiency cohort study. BMC Nephrol.

[35] Orchard TJ, Chang YF, Ferrell RE, Petro N, Ellis DE (2002). Nephropathy in type 1 diabetes: a manifestation of insulin resistance and multiple genetic susceptibilities? Further evidence from the Pittsburgh Epidemiology of Diabetes Complication Study. Kidney Int.

[36] Pilz S, Rutters F, Nijpels G, Stehouwer CDA, Hojlund K, Nolan JJ (2014). Insulin sensitivity and albuminuria: the RISC study. Diabetes Care.

[37] Matsumoto K, Sera Y, Abe Y, Ueki Y, Tominaga T, Miyake S (2003). Inflammation and insulin resistance are independently related to all-cause of death and cardiovascular events in Japanese patients with type 2 diabetes mellitus. Atherosclerosis..

[38] Zuliani G, Morieri ML, Volpato S, Maggio M, Cherubini A, Francesconi D (2014). Insulin resistance and systemic inflammation, but not metabolic syndrome phenotype, predict 9 years mortality in older adults. Atherosclerosis..

[39] Lee DY, Rhee EJ, Chang Y, Sohn CI, Shin HC, Ryu S (2018). Impact of systemic inflammation on the relationship between insulin resistance and all-cause and cancer-related mortality. Metabolism..

[40] Nyström T, Holzmann MJ, Eliasson B, Svensson AM, Kuhl J, Sartipy U (2017). Estimated glucose disposal rate and long-term survival in type 2 diabetes after coronary artery bypass grafting. Heart Vessel.

