# Supplementary material for: Insulin resistance, diabetic kidney disease, and all-cause mortality in individuals with type 2 diabetes: a prospective cohort study
Source: BMC Med. 2021 Mar 15;19:66. doi: 10.1186/s12916-021-01936-3 (PMC7962330; doi:10.1186/s12916-021-01936-3)
Supplement: Supplementary file 1 — Additional file 1: Figure S1. Scatterplot of clamp-derived GDR and eGDR (A) or HOMA-IR (B) in 140 individuals with T2D. [file 12916_2021_1936_MOESM1_ESM.doc]

**
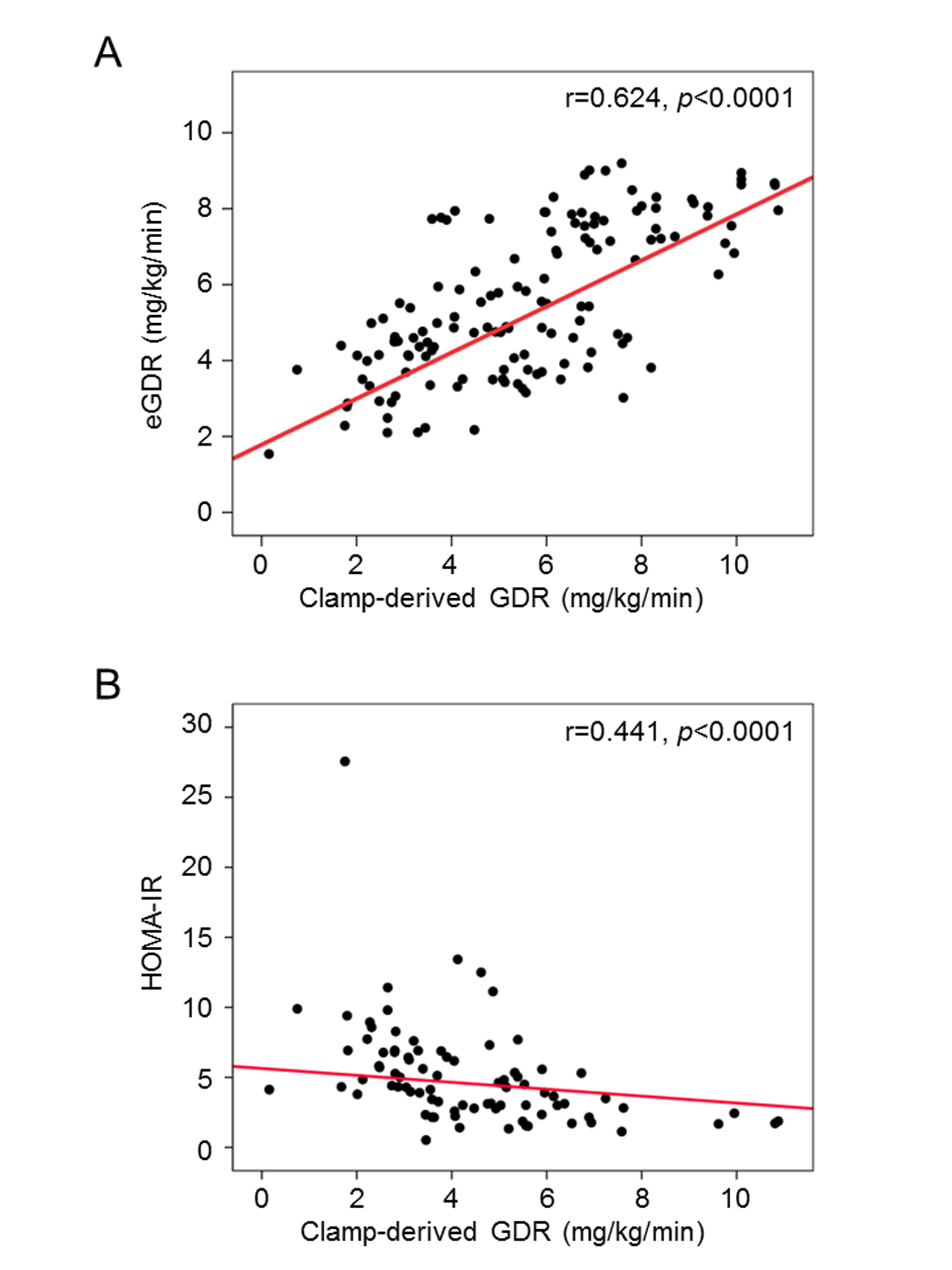
**

**Figure S1.** Scatterplot of clamp-derived GDR and eGDR (A) or HOMA-IR (B) in 140 individuals with T2D. Pearson correlation coefficients and correspondent *p* values are reported. GDR = glucose disposal rate; eGDR = estimated GDR; HOMA-IR = Homeostasis Model Assessment – Insulin Resistance; T2D =type 2 diabetes.
