# Supplementary material for: Insulin resistance, diabetic kidney disease, and all-cause mortality in individuals with type 2 diabetes: a prospective cohort study
Source: BMC Med. 2021 Mar 15;19:66. doi: 10.1186/s12916-021-01936-3 (PMC7962330; doi:10.1186/s12916-021-01936-3)
Supplement: Supplementary file 2 — Additional file 2: Table S1. Binary logistic regression analyses of kidney parameters (albuminuria categories, eGFR categories, and DKD phenotypes as dependent variables) with eGDR tertiles as covariate adjusted for confounders*. [file 12916_2021_1936_MOESM2_ESM.doc]

**Table S1.** Binary logistic regression analyses of kidney parameters (albuminuria categories, eGFR categories, and DKD phenotypes as dependent variables) with eGDR tertiles as covariate adjusted for confounders*.

|  | **OR** | **95% CI** | ***p*** |
| --- | --- | --- | --- |
| **Microalbuminuria (n=3,465) vs normoalbuminuria (n=11,460)** | | | <0.0001 |
| **T1** | 1 |  |  |
| **T2** | 1.349 | 1.218-1.493 | <0.0001 |
| **T3** | 1.943 | 1.757-2.148 | <0.0001 |
| **Macroalbuminuria (n=731) vs normoalbuminuria (n=11,460)** | | | <0.0001 |
| **T1** | 1 |  |  |
| **T2** | 1.463 | 1.154-1.855 | 0.002 |
| **T3** | 2.666 | 2.134-3.330 | <0.0001 |
| **eGFR 60-89 (n=7174) vs >90 (n=5,776) ml·min^-1^·1.73m^-2^** | | | <0.0001 |
| **T1** | 1 |  |  |
| **T2** | 1.159 | 1.052-1.278 | 0.003 |
| **T3** | 1.258 | 1.140-1.388 | <0.0001 |
| **eGFR 30-59 (n=2,427) vs >90 (n=5,776) ml·min^-1^·1.73m^-2^** | | | <0.0001 |
| **T1** | 1 |  |  |
| **T2** | 1.377 | 1.172-1.618 | <0.0001 |
| **T3** | 1.727 | 1.468-2.033 | <0.0001 |
| **eGFR<30 (n=279) vs >90 (n=5,776) ml·min^-1^·1.73m^-2^** | | | 0.016 |
| **T1** | 1 |  |  |
| **T2** | 1.096 | 0.728-1.650 | 0.661 |
| **T3** | 1.683 | 1.131-2.503 | 0.010 |
| **Albuminuric DKD with preserved eGFR (n=2,966) vs no DKD (n=9,984)** | | | <0.0001 |
| **T1** | 1 |  |  |
| **T2** | 1.438 | 1.287-1.607 | <0.0001 |
| **T3** | 2.387 | 2.145-2.656 | <0.0001 |
| **Nonalbuminuric DKD (n=1,476) vs no DKD (n=9,984)** | | | <0.0001 |
| **T1** | 1 |  |  |
| **T2** | 1.313 | 1.131-1.525 | <0.0001 |
| **T3** | 1.811 | 1.553-2.110 | <0.0001 |
| **Albuminuric DKD with reduced eGFR (n=1,230) vs no DKD (n=9,984)** | | | <0.0001 |
| **T1** | 1 |  |  |
| **T2** | 1.508 | 1.271-1.789 | <0.0001 |
| **T3** | 2.277 | 1.918-2.702 | <0.0001 |

* Adjusted for age, gender, smoking habits, diabetes duration, dyslipidaemia, non-advanced and advanced DR, prior CVD, cancer, and albuminuria (for eGFR categories) or eGFR (for albuminuria categories). eGFR = estimated glomerular filtration rate; DKD = diabetic kidney disease; eGDR = estimated glucose disposal rate; OR = odds ratio; CI = confidence interval; DR = diabetic retinopathy; CVD = cardiovascular disease.
