# Supplementary material for: Insulin resistance, diabetic kidney disease, and all-cause mortality in individuals with type 2 diabetes: a prospective cohort study
Source: BMC Med. 2021 Mar 15;19:66. doi: 10.1186/s12916-021-01936-3 (PMC7962330; doi:10.1186/s12916-021-01936-3)
Supplement: Supplementary file 3 — Additional file 3: Figure S2. Cumulative survival by Kaplan Meier analysis according to eGDR tertiles. Numbers (percentages) of death are shown for each tertile. [file 12916_2021_1936_MOESM3_ESM.doc]

**
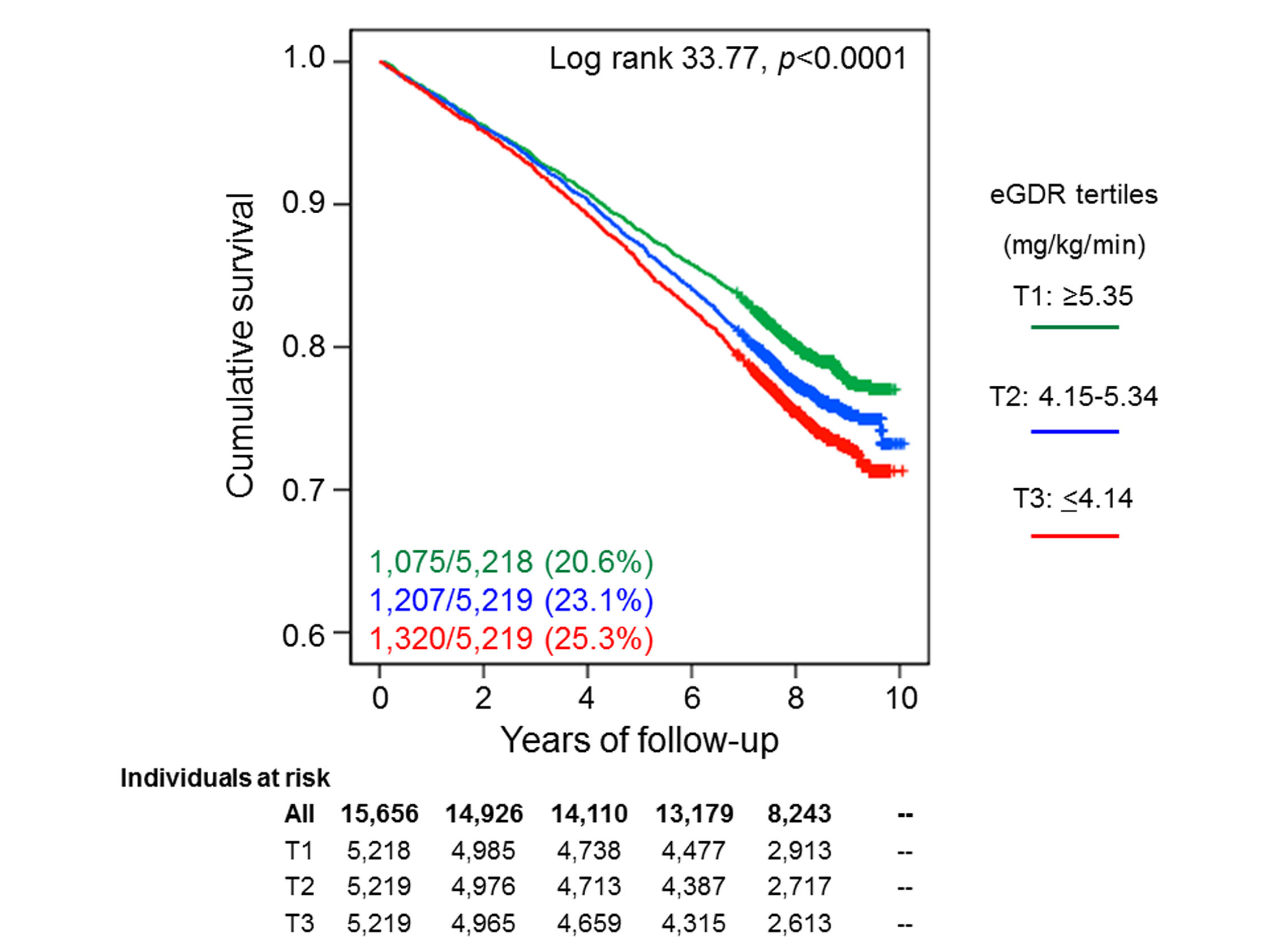
**

**Figure S2.** Cumulative survival by Kaplan Meier analysis according to eGDR tertiles. Numbers (percentages) of death are shown for each tertile. eGDR = estimated glucose disposal rate.
