# Supplementary material for: Insulin resistance, diabetic kidney disease, and all-cause mortality in individuals with type 2 diabetes: a prospective cohort study
Source: BMC Med. 2021 Mar 15;19:66. doi: 10.1186/s12916-021-01936-3 (PMC7962330; doi:10.1186/s12916-021-01936-3)
Supplement: Supplementary file 4 — Additional file 4: Table S2. Survival analysis by Cox proportional hazards regression according to eGDR tertiles, adjusted for age and gender (Model 1) and for age and gender plus albuminuria and eGFR categories (Model 2) or DKD phenotypes (Model 3). [file 12916_2021_1936_MOESM4_ESM.doc]

**Table S2.** Survival analysis by Cox proportional hazards regression according to eGDR tertiles, adjusted for age and gender (*Model 1*) and for age and gender plus albuminuria and eGFR categories (*Model 2*) or DKD phenotypes (*Model 3*).

|  | **HR** | **95% CI** | ***p*** |
| --- | --- | --- | --- |
| ***Model 1*** |  |  |  |
| **Male gender** | 1.518 | 1.419-1.624 | <0.0001 |
| **Age, years** | 1.100 | 1.096-1.105 | <0.0001 |
| **eGDR tertiles** |  |  | <0.0001 |
| **T1** | 1 |  |  |
| **T2** | 0.978 | 0.901-1.062 | 0.602 |
| **T3** | 1.347 | 1.242-1.460 | <0.0001 |
| ***Model 2*** |  |  |  |
| **Male gender** | 1.442 | 1.345-1.545 | <0.0001 |
| **Age, years** | 1.089 | 1.084-1.093 | <0.0001 |
| **Albuminuria categories** |  |  | <0.0001 |
| **A1 (normoalbuminuria)** | 1 |  |  |
| **A2 (microalbuminuria)** | 1.420 | 1.317-1.530 | <0.0001 |
| **A3 (macroalbuminuria)** | 2.143 | 1.901-2.416 | <0.0001 |
| **eGFR categories** |  |  | <0.0001 |
| **G1 (>90 ml·min^-1^·1.73m^-2^)** | 1 |  |  |
| **G2 (60-89 ml·min^-1^·1.73m^-2^)** | 1.023 | 0.929-1.127 | 0.641 |
| **G3 (30-59 ml·min^-1^·1.73m^-2^)** | 1.560 | 1,396-1.742 | <0.0001 |
| **G4-5 (<30 ml·min^-1^·1.73m^-2^)** | 2.577 | 2.158-3.077 | <0.0001 |
| **eGDR tertiles** |  |  | <0.0001 |
| **T1** | 1 |  |  |
| **T2** | 0.919 | 0.846-0.998 | 0.045 |
| **T3** | 1.180 | 1.087-1.281 | <0.0001 |
| ***Model 3*** |  |  |  |
| **Male gender** | 1.444 | 1.348-1.547 | <0.0001 |
| **Age, years** | 1.088 | 1.083-1.092 | <0.0001 |
| **DKD phenotypes** |  |  | <0.0001 |
| **No DKD** | 1 |  |  |
| **Albuminuric DKD with preserved eGFR** | 1.608 | 1.474-1.755 | <0.0001 |
| **Nonalbuminuric DKD** | 1.727 | 1.564-1.906 | <0.0001 |
| **Albuminuric DKD with reduced eGFR** | 2.587 | 2.351-2.846 | <0.0001 |
| **eGDR tertiles** |  |  | <0.0001 |
| **T1** | 1 |  |  |
| **T2** | 0.919 | 0.846-0.998 | 0.044 |
| **T3** | 1.189 | 1.095-1.290 | <0.0001 |

eGDR = estimated glucose disposal rate; eGFR = estimated glomerular filtration rate; DKD = diabetic kidney disease; HR = hazard ratio; CI = confidence interval.
