# Supplementary material for: Insulin resistance, diabetic kidney disease, and all-cause mortality in individuals with type 2 diabetes: a prospective cohort study
Source: BMC Med. 2021 Mar 15;19:66. doi: 10.1186/s12916-021-01936-3 (PMC7962330; doi:10.1186/s12916-021-01936-3)
Supplement: Supplementary file 5 — Additional file 5: Table S3. Survival analysis by Cox proportional hazards regression according to eGDR tertiles, adjusted for age, gender, albuminuria and eGFR categories (Model 4) or DKD phenotypes (Model 5), plus multiple confounders*. [file 12916_2021_1936_MOESM5_ESM.doc]

**Table S3.** Survival analysis by Cox proportional hazards regression according to eGDR tertiles, adjusted for age, gender, albuminuria and eGFR categories (*Model 4*) or DKD phenotypes (*Model 5*), plus multiple confounders*.

|  | **HR** | **95% CI** | ***p*** |
| --- | --- | --- | --- |
| ***Model 4*** |  |  |  |
| **Male gender** | 1.319 | 1.222-1.419 | <0.0001 |
| **Age, years** | 1.086 | 1.081-1.091 | <0.0001 |
| **Albuminuria categories** |  |  | <0.0001 |
| **A1 (normoalbuminuria)** | 1 |  |  |
| **A2 (microalbuminuria)** | 1.308 | 1.213-1.412 | <0.0001 |
| **A3 (macroalbuminuria)** | 1.941 | 1.719-2.192 | <0.0001 |
| **eGFR categories** |  |  | <0.0001 |
| **G1 (>90 ml·min^-1^·1.73m^-2^)** | 1 |  |  |
| **G2 (60-89 ml·min^-1^·1.73m^-2^)** | 0.996 | 0.904-1.097 | 0.928 |
| **G3 (30-59 ml·min^-1^·1.73m^-2^)** | 1.440 | 1.287-1.610 | <0.0001 |
| **G4-5 (<30 ml·min^-1^·1.73m^-2^)** | 2.196 | 1.836-2.628 | <0.0001 |
| **Smoking status** |  |  | <0.0001 |
| **Never** | 1 |  |  |
| **Former** | 1.052 | 0.974-1.137 | 0.198 |
| **Current** | 1.276 | 1.154-1.411 | <0.0001 |
| **Diabetes duration, years** | 1.004 | 1.001-1.008 | 0.012 |
| **Dyslipidaemia** | 0.692 | 0.639-0.749 | <0.0001 |
| **DR grade** |  |  | <0.0001 |
| **No** | 1 |  |  |
| **Non-advanced** | 1.123 | 1.022-1.233 | 0.016 |
| **Advanced** | 1.304 | 1.180-1.441 | <0.0001 |
| **Any CVD** | 1.594 | 1.485-1.710 | <0.0001 |
| **Any cancer** | 1.789 | 1.612-1.985 | <0.0001 |
| **eGDR tertiles** |  |  | <0.0001 |
| **T1** | 1 |  |  |
| **T2** | 0.904 | 0.832-0.982 | 0.017 |
| **T3** | 1.140 | 1.049-1.238 | 0.002 |
| ***Model 5*** |  |  |  |
| **DKD phenotypes** |  |  | <0.0001 |
| **No DKD** | 1 |  |  |
| **Albuminuric DKD with preserved eGFR** | 1.485 | 1.360-1.622 | <0.0001 |
| **Nonalbuminuric DKD** | 1.634 | 1.480-1.804 | <0.0001 |
| **Albuminuric DKD with reduced eGFR** | 2.190 | 1.986-2.415 | <0.0001 |
| **eGDR tertiles** |  |  | <0.0001 |
| **T1** | 1 |  |  |
| **T2** | 0.898 | 0.826-0.976 | 0.011 |
| **T3** | 1.140 | 1.049-1.239 | 0.002 |

* CVD risk factors (smoking habits, diabetes duration, and dyslipidaemia) and complications/comorbidities (DR grade, prior CVD, and cancer). eGDR = estimated glucose disposal rate; eGFR = estimated glomerular filtration rate; HR = hazard ratio; CI = confidence interval; DR = diabetic retinopathy; DKD = diabetic kidney disease; CVD = cardiovascular disease.
