# Supplementary material for: Insulin resistance, diabetic kidney disease, and all-cause mortality in individuals with type 2 diabetes: a prospective cohort study
Source: BMC Med. 2021 Mar 15;19:66. doi: 10.1186/s12916-021-01936-3 (PMC7962330; doi:10.1186/s12916-021-01936-3)
Supplement: Supplementary file 6 — Additional file 6: Figure S3. Cox proportional hazards regression, unadjusted (A), and adjusted by age and gender (B; model 1), plus albuminuria and eGFR categories (C; model 2) plus multiple confounders* (D; model 4), according to tertiles of eGDR calculated using measured waist circumference. HRs (95% CI) for mortality are shown for each tertile. [file 12916_2021_1936_MOESM6_ESM.doc]

**
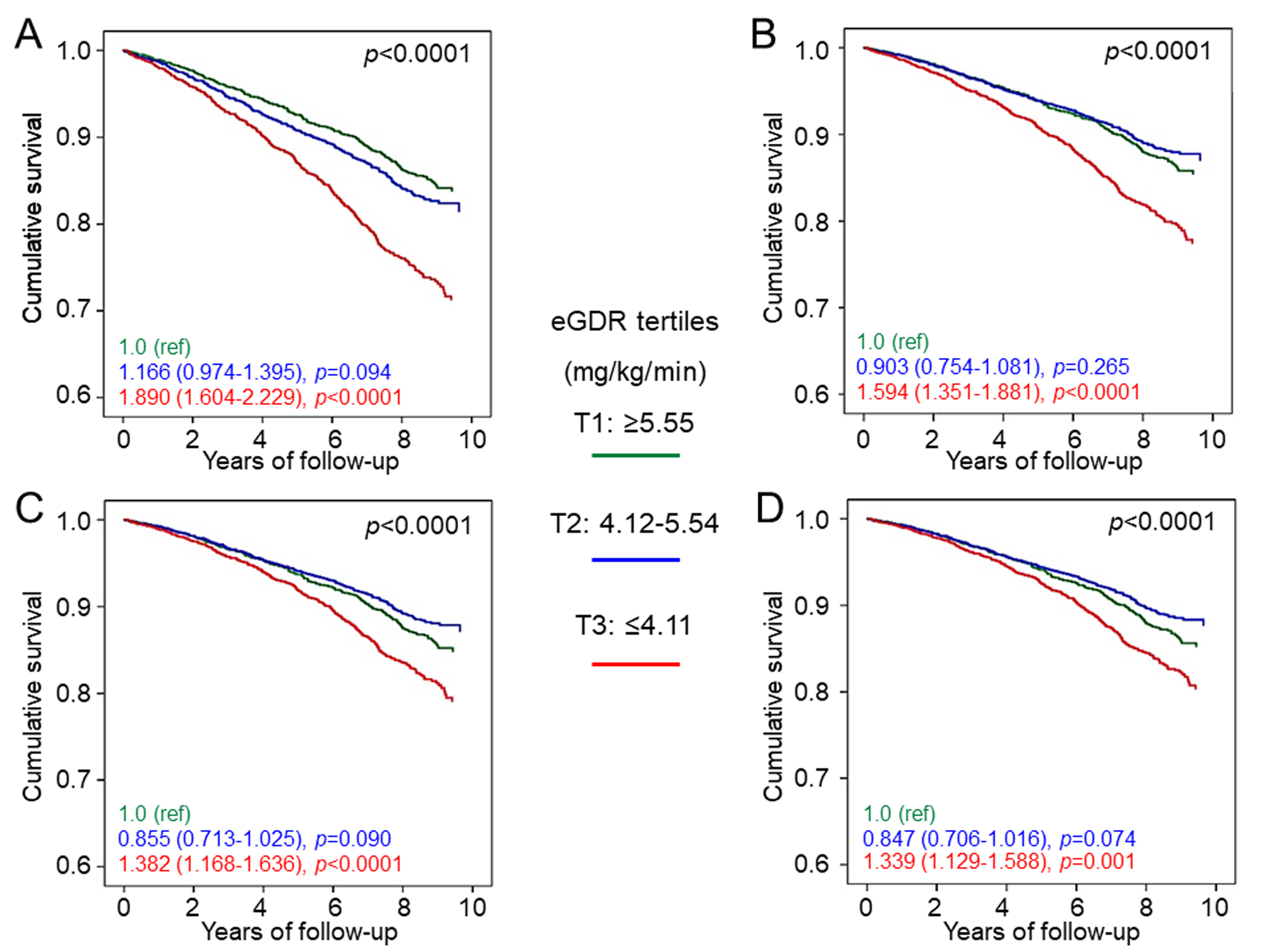
**

**Figure S3.** Cox proportional hazards regression, unadjusted (A), and adjusted by age and gender (B; model 1), plus albuminuria and eGFR categories (C; model 2) plus multiple confounders* (D; model 4), according to tertiles of eGDR calculated using measured waist circumference. HRs (95% CI) for mortality are shown for each tertile. * CVD risk factors (smoking habits, diabetes duration, and dyslipidaemia) and complications/comorbidities (DR grade, prior CVD, and cancer). eGFR = estimated glomerular filtration rate; eGDR = estimated glucose disposal rate; HR = hazard ratio; CI = confidence interval; CVD = cardiovascular disease; DR = diabetic retinopathy.
