# Supplementary material for: Insulin resistance, diabetic kidney disease, and all-cause mortality in individuals with type 2 diabetes: a prospective cohort study
Source: BMC Med. 2021 Mar 15;19:66. doi: 10.1186/s12916-021-01936-3 (PMC7962330; doi:10.1186/s12916-021-01936-3)
Supplement: Supplementary file 7 — Additional file 7: Table S4. Survival analysis by Cox proportional hazards regression according to eGDR tertiles in subgroups. [file 12916_2021_1936_MOESM7_ESM.doc]

**Table S4.** Survival analysis by Cox proportional hazards regression according to eGDR tertiles in subgroups.

|  | **HR** | **95% CI** | ***p* value** | ***p* value for interaction** |
| --- | --- | --- | --- | --- |
| **Age** |  |  |  | 0.001 |
| **Below median value (n=7,829)** | |  | <0.0001 |  |
| **T1** | 1 |  |  |  |
| **T2** | 0.888 | 0.737-1.071 | 0.214 |  |
| **T3** | 1.339 | 1.132-1.584 | 0.001 |  |
| **Above median value (n=7,827)** | |  | <0.0001 |  |
| **T1** | 1 |  |  |  |
| **T2** | 0.915 | 0.834-1.004 | 0.061 |  |
| **T3** | 1.104 | 1.002-1.216 | 0.045 |  |
| **Gender** |  |  |  | 0.533 |
| **Males (n=8,902)** |  |  | <0.0001 |  |
| **T1** | 1 |  |  |  |
| **T2** | 0.888 | 0.799-0.986 | 0.027 |  |
| **T3** | 0.172 | 1.054-1.303 | 0.003 |  |
| **Females (n=6,754)** |  |  | 0.052 |  |
| **T1** | 1 |  |  |  |
| **T2** | 0.918 | 0.801-1.052 | 0.217 |  |
| **T3** | 1.072 | 0.936-1.227 | 0.316 |  |
| **Prior CVD** | |  |  | 0.368 |
| **No (n=12,036)** | |  | 0.001 |  |
| **T1** | 1 |  |  |  |
| **T2** | 0.933 | 0.841-1.035 | 0.188 |  |
| **T3** | 1.129 | 1.018-1.253 | 0.022 |  |
| **Yes (n=3,620)** | |  | <0.0001 |  |
| **T1** | 1 |  |  |  |
| **T2** | 0.852 | 0.740-0.980 | 0.025 |  |
| **T3** | 1.119 | 0.972-1.287 | 0.117 |  |

Data are adjusted for age, gender, albuminuria and eGFR categories, CVD risk factors (smoking habits, diabetes duration, and dyslipidaemia) and complications/comorbidities (DR grade, prior CVD, and cancer). eGDR = estimated glucose disposal rate; HR = hazard ratio; CI = confidence interval; eGFR = estimated glomerular filtration rate; CVD = cardiovascular disease; DR = diabetic retinopathy.
